# Protective effect of *Vigna unguiculata* extract against aging and neurodegeneration

**DOI:** 10.18632/aging.104069

**Published:** 2020-10-05

**Authors:** Farida Tripodi, Linda Lombardi, Lorenzo Guzzetti, Davide Panzeri, Riccardo Milanesi, Manuela Leri, Monica Bucciantini, Cristina Angeloni, Daniela Beghelli, Silvana Hrelia, Giada Onorato, Elia Di Schiavi, Ermelinda Falletta, Simona Nonnis, Gabriella Tedeschi, Massimo Labra, Paola Coccetti

**Affiliations:** 1 Department of Biotechnology and Biosciences, University of Milano-Bicocca, Milano, Italy; 2 Department of Experimental and Clinical Biomedical Sciences, University of Firenze, Firenze, Italy; 3 Department of Neuroscience, Psychology, Drug Research and Child Health, University of Firenze, Firenze, Italy; 4 School of Pharmacy, University of Camerino, Camerino, Italy; 5 School of Biosciences and Veterinary Medicine, University of Camerino, Camerino, Italy; 6 Department for Life Quality Studies, Alma Mater Studiorum, University of Bologna, Rimini, Italy; 7 Institute of Biosciences and BioResources (IBBR), CNR, Naples, Italy; 8 Department of Chemistry, University of Milano, Milano, Italy; 9 Department of Veterinary Medicine (DIMEVET), University of Milano, Milano, Italy

**Keywords:** *Saccharomyces cerevisiae*, *Drosophila melanogaster*, *Caenorhabditis elegans*, human α-synuclein, Parkinson’s disease (PD)

## Abstract

Aging and age-related neurodegeneration are among the major challenges in modern medicine because of the progressive increase in the number of elderly in the world population. Nutrition, which has important long-term consequences for health, is an important way to prevent diseases and achieve healthy aging. The beneficial effects of *Vigna unguiculata* on metabolic disorders have been widely documented. Here, we show that an aqueous extract of *V. unguiculata* beans delays senescence both in *Saccharomyces cerevisiae* and *Drosophila melanogaster*, in a Snf1/AMPK-dependent manner. Consistently, an increased expression of FOXO, SIRT1, NOTCH and heme oxygenase (HO) genes, already known to be required for the longevity extension in *D. melanogaster*, is also shown. Preventing α-synuclein self-assembly is one of the most promising approaches for the treatment of Parkinson’s disease (PD), for which aging is a risk factor. *In vitro* aggregation of α-synuclein, its toxicity and membrane localization in yeast and neuroblastoma cells are strongly decreased in the presence of bean extract. In a *Caenorhabditis elegans* model of PD, *V. unguiculata* extract substantially reduces the number of the age-dependent degeneration of the cephalic dopaminergic neurons. Our findings support the role of *V. unguiculata* beans as a functional food in age-related disorders.

## INTRODUCTION

Nutrients and their metabolites control energy balance, enzymatic activities and genome stability throughout the lifecycle. It is an unequivocal statement that nutritional deficiency as well as excess contribute to the aging process. Dietary restriction is known as the most effective longevity intervention ranging from yeast to primates [1–6]. Several results have suggested new roles of key nutrients in the protection against aging and age-related disorders [5, 7]. Thus, there is an increasing interest in nutrition as a way both to prevent diseases and to reach healthy aging.

Much of the current knowledge on the molecular mechanisms of aging comes from lifespan studies on short-lived model organisms, such as the budding yeast *Saccharomyces cerevisiae*, *Drosophila melanogaster* and *Caenorhabditis elegans* [8]. Specifically, AMPK (AMP-activated protein kinase), IGF (insulin-like growth factor) and TORC1 (target of rapamycin kinase complex 1) signaling pathways play key functions in regulating aging [9–11].

Neurodegenerative diseases, characterized by aberrant aggregates of the presynaptic protein α-synuclein, are collectively referred to as synucleinopathies, the second most common group of neurodegenerative diseases [12–14]. One of the most common synucleinopathies is Parkinson’s disease (PD) and autosomal dominant forms of PD have been linked to mutations in α-synuclein. In PD patients, neurodegeneration is found predominantly in dopaminergic neurons. Despite the advances in the study of these pathologies, the detailed molecular mechanism of neuronal degeneration is still largely unknown. Several studies underline the relevant role of cellular models for a better understanding of the molecular regulation of human pathologies [15]. As such, budding yeast has been extensively employed in models of synucleinopathies [16–19]. In addition, an age-related degeneration of dopaminergic neurons has been shown in wild-type *C. elegans* [20]. Interestingly, neuronal and dendritic loss are accelerated and more severe when human α-synuclein is expressed in dopaminergic neurons in *C. elegans* [21].

*Vigna unguiculata* (L.) Walp. or cowpea is the most relevant *Vigna* species for human food. It is cultivated in tropical and subtropical zones of the world, including Africa, Asia, Latin America and also in some Mediterranean countries [22, 23]. Cowpea seeds are a good source of proteins, which mainly consist of globulins (vicilins or 7S globulins) and, to a lesser extent, albumins, glutelins and prolamins [24]. From a nutritional point of view, there is a high ratio of essential-to-non-essential amino acids, which is over 50%, suggesting the potential capacity of cowpea to cover human nutritional requirements [25, 26]. Moreover, bioactive peptides with antioxidant activity are successfully obtained from enzymatic proteolysis of cowpea proteins, indicating also its potentiality as a functional food [24]. In comparison with other legumes, cowpea has a low-fat content with high level of unsaturated fatty acids and is also characterized by a high proportion of carbohydrates (mainly dietary fibers and resistant starch) [24]. Apart from the relevant source of essential macronutrients, cowpea also constitutes an interesting source of micronutrients [27].

All these features, together with the presence of minerals (calcium, iron and zinc) and phytochemicals, such as phenolic compounds, are attracting the attention of consumers and researchers, also because of its beneficial properties for health, including anti-diabetic, anti-cancer, anti-hyperlipidemic, anti-inflammatory and anti-hypertensive properties [28].

The aim of the present study is to investigate whether *V. unguiculata* also has anti-aging and neuroprotective effects, exploiting different model organisms to address complementary aspects. We show that an aqueous extract from *V. unguiculata* beans increases lifespan in yeast cells, being dependent on Snf1/AMPK (sucrose-non-fermenting/AMP-activated protein kinase) and Ras/PKA (Rat sarcoma/protein kinase A) pathways. Its pro-longevity feature is also confirmed on the multicellular organism *D. melanogaster*, which is consistent with the increased expression of AMPK-dependent genes associated with fly lifespan extension. Cowpea extract is able to significantly reduce the aggregation of α-synuclein *in vitro* and to attenuate its toxicity both in yeast and neuroblastoma cells. Remarkably, in a nematode model expressing human α-synuclein, the age-dependent degeneration of the dopaminergic neurons is strongly reduced under chronic treatments with *V. unguiculata* extract.

## RESULTS

### *Vigna unguiculata* extract extends lifespan in yeast cells

**Figure 1:**
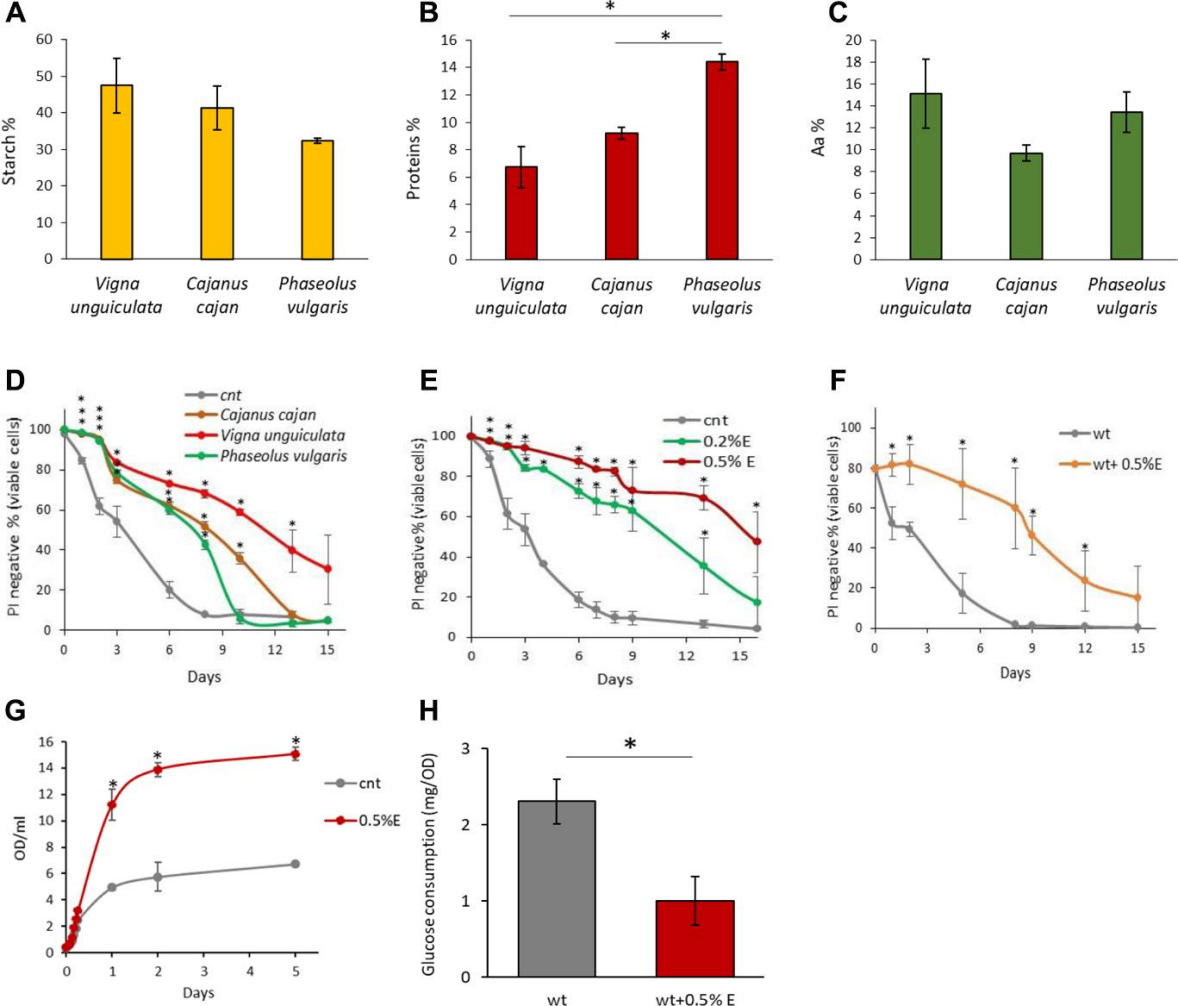
**Chemical properties of bean extracts.** (**A**) Starch content, (**B**) protein content and (**C**) amino acid content in *V. unguiculata*, *C. cajan* and *P. vulgaris* extracts. *p<0.05. (**D**) CLS of yeast cells grown in the absence or presence of 0.2% *V. unguiculata*, *C. cajan* and *P. vulgaris* extracts. *p<0.05 relative to control cells. (**E**) CLS of yeast cells grown in SD medium containing 2% glucose in the absence or presence of 0.2% or 0.5% *V. unguiculata* extract, added in exponential phase of growth. *p<0.05 relative to control cells. (**F**) CLS of yeast cells grown in SD medium containing 2% glucose in the absence or presence of 0.5% *V. unguiculata* extract, added to cells in stationary phase (and not in exponential phase, as in the other experiments). *p<0.05 relative to control cells. (**G**) Growth curves of yeast cells grown in SD medium containing 2% glucose in the absence or presence of 0.5% extract *V. unguiculata*. *p<0.05 relative to control cells. (**H**) Glucose consumption (mg/OD) of yeast cells grown in SD medium in the absence or presence of 0.5% *V.* *unguiculata* extract, measured on growth media sampled at multiple time points during exponential phase of growth (0.2-2.5 OD/ml). *p<0.05 relative to control cells.

Considering the nutritional properties and positive effects for health of *Vigna unguiculata* [28], we investigated the composition of aqueous bean extracts from *V. unguiculata* in comparison with those obtained from *Cajanus cajan* L. and *Phaseolus vulgaris* L., originating from the Arusha area in Tanzania. *V. unguiculata* extract was characterized by a higher starch amount and less proteins compared to the extracts obtained from the other pulses, while the percentage of total amino acids was comparable among species (Figure 1A–1C). We also confirmed that cowpea seeds are a good source of amino acids (included the essential ones), as well as of unsaturated fatty acids (more abundant in comparison with the other two beans), confirming its nutritionally desirable features (Supplementary Tables 1, 2) [24].

To explore if these differences could have an impact on the longevity of yeast cells, exponentially growing cells were treated with 0.2% of the extracts from *V. unguiculata*, *C. cajan* or *P. vulgaris* beans and chronological lifespan was monitored by measuring the viability of the cultures throughout time. Although all the extracts increased longevity of yeast cells, the highest response was evident in the presence of *V. unguiculata* one, with a mean lifespan increasing from 3 days to about 9.5 days (Figure 1D, Table 1). On the basis of the above results, we decided to continue our analysis by using only the cowpea extract. A strong dose-response effect on yeast longevity was observed by increasing the concentration of *V. unguiculata* extract in the culture (from 0.2% to 0.5%), since it was able to extend the mean lifespan up to 16 days at the higher concentration (Figure 1E, Table 1). Its anti-aging properties were evident also when the extract was added to “aged” cells, *i.e.* after they had already entered the stationary phase (Figure 1F). Remarkably, cowpea extract not originating from Arusha maintained the same effect, letting us to suppose that the origin of the beans has no relevant impact on its anti-aging features (data not shown).

**Table 1:** Mean and maximal lifespan of wt cells grown in the presence of the indicated extracts.

**lifespan (days)**		
**wt strain**	**mean**	**maximal**
cnt (no extract)	3.05 ± 0.42	9.46 ± 1.47
0.2% *P. vulgaris*	6.42 ± 0.12	11.72 ± 0.05
0.2% *C. cajan*	7.74 ± 0.30	13.53 ± 0.32
0.2% *V. unguiculata*	9.55 ± 0.49	18.55 ± 0.64
0.5% *V. unguiculata*	16.03 ± 3.01	>20

Cell growth was then monitored in the presence of the highest concentration of the extract. Although the growth rate in the presence of 0.5% extract showed only a minor increase in exponential phase, the final biomass of the population was more than doubled in comparison with the control (Figure 1G). On the other hand, the consumption of glucose in the media during the exponential phase of growth was strongly reduced (more than 50%), suggesting that the presence of either starch or proteins induced a decrease of glucose uptake from the medium (Figure 1H). However, the lower glucose consumption in the presence of the extract have no effect on the experimental determination of CLS, which starts after carbon source exhaustion.

Importantly, the anti-aging effect of the extract was synergistic with caloric restriction, one of the most effective non-genetic interventions known to promote lifespan extension in several model organisms [10] (Supplementary Figure 1). Overall, the data presented indicate that cowpea aqueous extract extends chronological aging in yeast cells.

**Figure 2:**
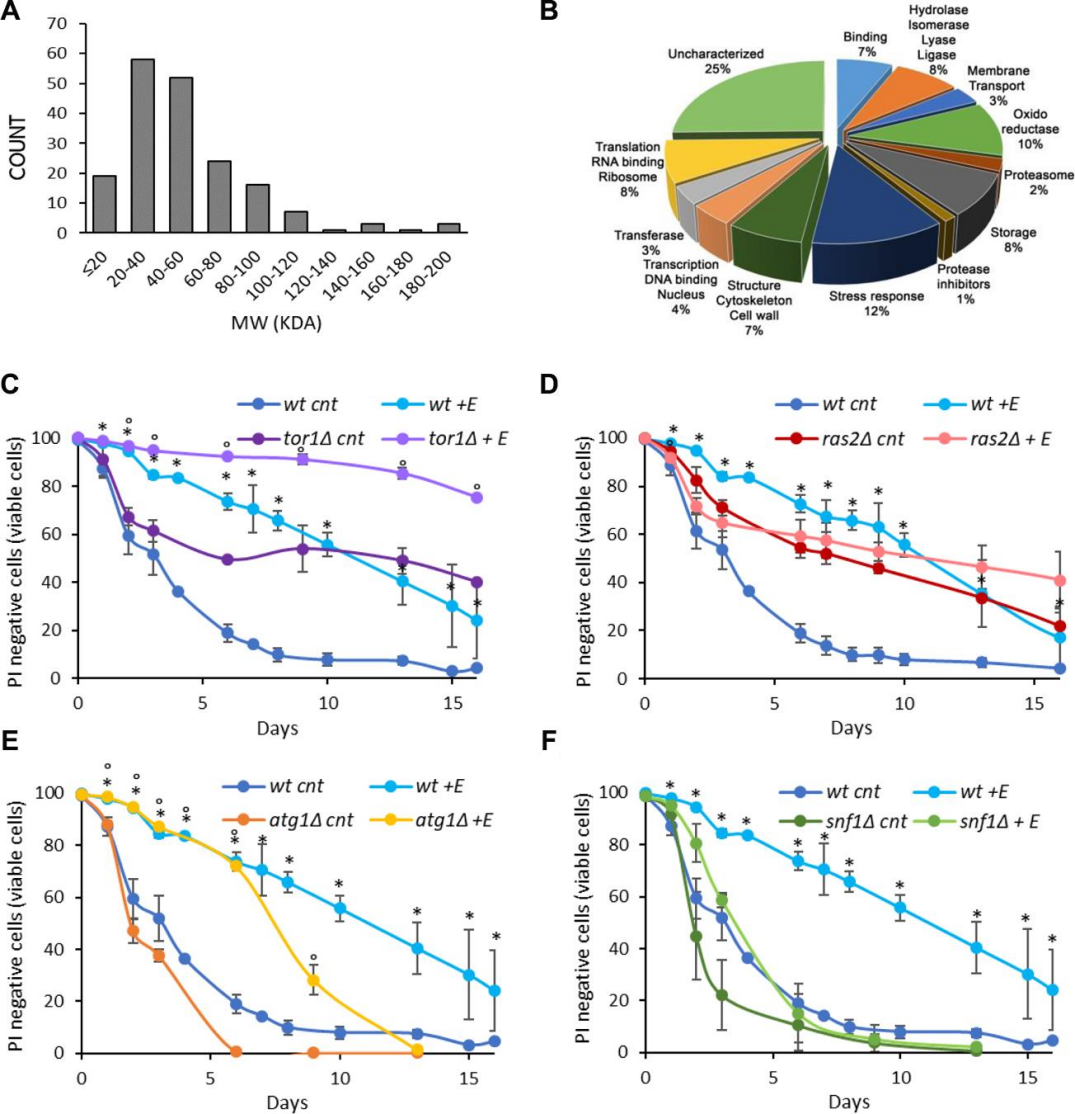
**Cowpea extract extends yeast lifespan.** (**A**, **B**) Analysis of *V. unguiculata* extract by mass spectrometry using a shotgun proteomic approach to identify all the proteins present in the sample. (A) MW distribution and (B) classification of the proteins identified. The data have been deposited to the ProteomeXchange Consortium via the PRIDE partner repository with the dataset identifier PXD017716. (**C**–**F**) CLS of wt and (**C**) *tor1Δ*, (**D**) *ras2Δ*, (**E**) *atg1Δ*, (**F**) *snf1Δ* cells, grown in SD medium containing 2% glucose in the absence or presence of 0.2% *V. unguiculata* extract. *p<0.05 relative to untreated wt cells, °p<0.05 relative to untreated mutant cells. Curves of wt untreated cells and treated with the extract were repeated in **C**–**F**.

To increase our knowledge on the composition of *V. unguiculata* extract we performed a proteomic analysis. Interestingly, we identified 174 proteins with a molecular weight ranging from 200 to less than 20 kDa, of which 10% are oxidoreductase, 12% are stress response proteins while 25% are still uncharacterized (Figure 2A, 2B).

The signaling pathways connected to longevity regulation are well known in yeast. Among them, the Snf1/AMPK (sucrose-non fermenting/AMP-activated protein kinase) and the autophagic pathways are anti-aging pathways, while the Ras2/PKA (Rat sarcoma/protein kinase A) and the TORC1 (target of rapamycin complex 1) pathways are pro-aging ones [8, 9, 29]. To identify through which of them the cowpea extract extended yeast chronological lifespan, we tested its effect on mutants bearing deletion in one of the aforementioned pathways (*snf1Δ*, *atg1Δ*, *ras2Δ*, *tor1Δ*) (Figure 2C–2F, Table 2). The anti-aging effect of 0.2% cowpea extract was still evident in *tor1Δ* and *atg1Δ* strains (Figure 2C, 2E, Table 2), while it was strongly reduced in *ras2Δ* and *snf1Δ* mutants (Figure 2D, 2F, Table 2), indicating that the Ras/PKA and the Snf1/AMPK pathways are involved in mediating the anti-aging effect of cowpea extract in yeast cells.

**Table 2:** Mean and maximal lifespan of mutant strains grown in the presence of 0.2% *V. unguiculata* extract. Data of wt cells were repeated for clarity.

**lifespan (days)**				
	**mean**		**maximal**	
**strain**	**cnt**	**0.2% E**	**cnt**	**0.2% E**
*wt*	3.05 ± 0.42	9.55 ± 0.49	9.46 ± 1.47	18.55 ± 0.64
*ras2Δ*	7.48 ± 0.40	10.48 ± 4.98	18.95 ± 1.34	>20
*tor1Δ*	5.87 ± 0.23	>20	18.5 ± 0.14	>20
*atg1Δ*	2.22 ± 0.10	7.35 ± 0.21	4.95 ± 0.21	11.45 ± 0.78
*snf1Δ*	2.0 ± 0.42	3.35 ± 0.21	4.8 ± 2.40	7.2 ± 1.56

### *Vigna unguiculata* extract supplementation extends lifespan in *Drosophila melanogaster*

**Figure 3:**
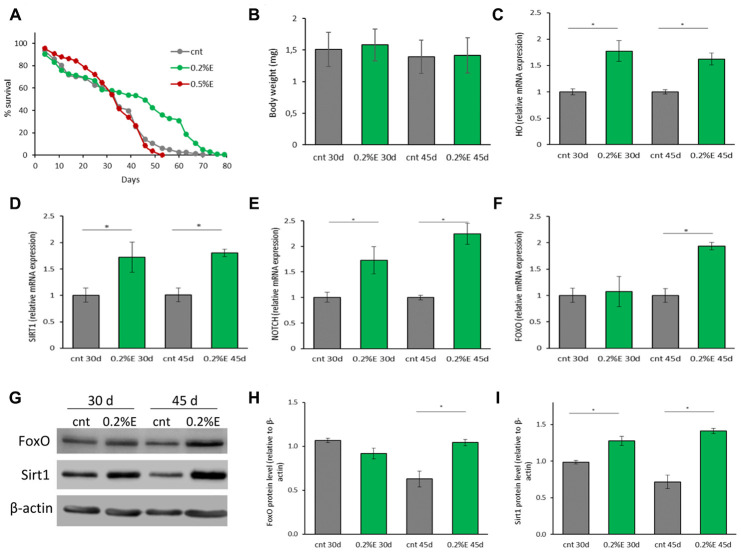
**Cowpea extract extends *D. melanogaster* lifespan.** (**A**) Survivorship of adult female *D. melanogaster*. Flies were supplemented with 0.2% and 0.5% bean extract lifelong. (**B**) Body weights of *D. melanogaster* supplemented with 0.2% bean extract. Flies were supplemented with 0.2% bean extract for 30 or 45 days. (**C**–**F**) Expression of genes related to longevity and oxidative stress. Flies were supplemented with 0.2% cowpea extract for 30 or 45 days. Total RNA was isolated and the mRNA levels of HO (**C**), SIRT1 (**D**), NOTCH (**E**), FOXO (**F**) were quantified using RT-PCR. (**G**) Western analysis using anti-FoxO and anti-Sirt1 antibodies on proteins extracts from flies supplemented with 0.2% cowpea extract for 30 or 45 days. (**H**–**I**) Densitometric analysis of FoxO and Sirt1 proteins. *p<0.05 with respect to the corresponding controls.

To investigate the pro-longevity effect of the cowpea extract also in a multicellular organism, female Canton S flies were lifelong supplemented with 0.2% or 0.5% *V. unguiculata* extract. A significant marked increase in mean lifespan was observed in flies supplemented with 0.2% bean extract in respect to controls (40.09±1.08 days *vs* 31.82±0.86 days; 25.99% increase), while mean lifespan of flies supplemented with 0.5% bean extract was comparable to that of control flies (Figure 3A). These data are only partially in agreement with the results obtained in yeast cells, where the 0.5% cowpea supplementation was more effective than the 0.2% one. Nevertheless, considering only the survivorship data obtained after a 3 weeks supplementation, a higher survival of flies supplemented with 0.5% in respect to both 0.2% supplement and control was observed (Supplementary Figure 2A, 2B).

To verify whether the increase of the mean lifespan in the presence of 0.2% extract was due to bean extract supplementation itself and not to CR induced by bean extract off-flavor, the body weights of flies were recorded at 30 and 45 days. No differences in fly body weights were observed, suggesting an equal food uptake in control and supplemented groups (Figure 3B).

To better clarify, at a molecular level, the positive effect of 0.2% cowpea extract supplementation on *D. melanogaster* lifespan, the expression of genes involved in preserving cellular homeostasis and longevity was investigated. Flies were supplemented with cowpea extract for 30 or 45 days and the expression of genes involved in aging-related signaling pathways (SIRT1 -sirtuin 1-, FOXO -Forkhead box O- and NOTCH) and antioxidant defense systems (HO - heme oxygenase and TRXR - thioredoxin reductase) were measured (Figure 3C–3F). Oxidative stress has been recognized to play a key role in aging [30]. The oxidative stress theory of aging speculates that the functional losses typical of elderly are associated with the accumulation of structural impairments caused by the oxidative damage to macromolecules [31]. HO expression was significantly up-regulated by cowpea extract supplementation after both 30 and 45 days (Figure 3C), while TRXR was not influenced at all (data not shown), suggesting that *V. unguiculata* extract partially modulates the endogenous antioxidant defense system.

SIRT1, a member of the class III NAD^+^-dependent histone deacetylases (HDACs) has been implicated in the extension of longevity in *D. melanogaster* [32]. SIRT1 expression was significantly up-regulated in flies supplemented with cowpea extract after both 30 and 45 days (Figure 3D). Remarkably, also NOTCH expression increased at both time points (Figure 3E), in accordance with findings showing that SIRT1 is a positive modulator of NOTCH [33]. FOXO is a fundamental transcriptional regulator of the insulin pathway modulating growth and proliferation and its increase has been associated with extension of flies lifespan [34]. Although FOXO expression after 30 days of supplementation with *V. unguiculata* was the same as in control flies, cowpea extract triggered a significant up-regulation of FOXO expression at 45 days (Figure 3F). In agreement with the gene expression, the level of FoxO and Sirt1 proteins increased in flies supplemented with cowpea extract (Figure 3G–3I).

Thus, the aging-related signaling pathways of SIRT1, FOXO and NOTCH are involved in mediating the effect of cowpea extract in fruit flies.

### *Vigna unguiculata* extract reduces both α-synuclein toxicity and aggregation *in vitro*

Extensive literature reports the fibrillation-inhibiting effects of plant extracts, including those consumed as part of a healthy diet [35, 36] and others found in traditional medicine [37–40].

**Figure 4:**
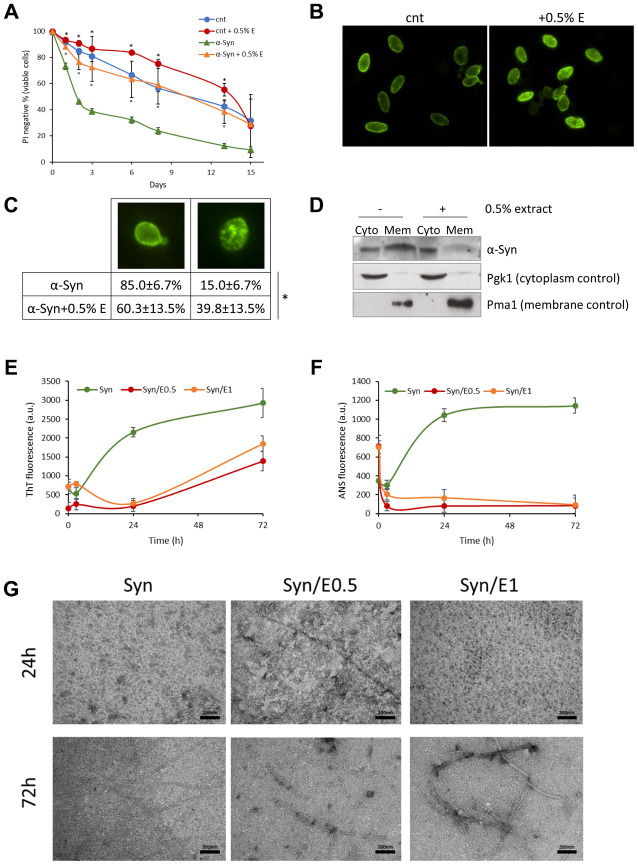
**Cowpea extract reduces α-synuclein toxicity and aggregation.** (**A**) CLS of yeast cells bearing pYX242 empty vector or pYX242-SNCA plasmid grown in SD medium containing 2% glucose in the absence or presence of 0.5% *V. unguiculata* extract. *p<0.05 relative to untreated cells bearing the empty vector, °p<0.05 relative to untreated α-synuclein expressing cells. (**B**, **C**) Immunofluorescence showing localization of α-synuclein in cells untreated or treated for 1 day with 0.5% *V. unguiculata* extract. The percentage of cells with α-synuclein localized in the cellular membrane is shown in (**C**). *p<0.05. (**D**) Western analysis using anti-α-synuclein antibody on cytolpasmic and membrane fractions isolated from wt [pYX242-SNCA] cells after 1-day treatment with 0.5% *V. unguiculata* extract. Pgk1 was used as cytoplasmic marker, Pma1 as membrane marker. (**E**, **F**) α-synuclein aggregation process followed by ThT fluorescence (**E**) and ANS binding (**F**) assays. (**G**) TEM pictures taken from α-synuclein aggregation mixture after 24 h and 72 h of incubation in the absence or in the presence of cowpea extract at molar ratio protein:extract 1:0.5 (E0.5) and 1:1 (E1); scale bars are shown.

α-Synuclein is a presynaptic protein associated with the pathophysiology of synucleinopathies, including Parkinson’s disease [12–14], and budding yeast has been extensively employed in models of synucleinopathies [16]. Thus, the effect of *V. unguiculata* extract on the longevity of yeast cells over-expressing the human α-synuclein [41] was evaluated. Interestingly, the addition of cowpea extract to exponentially growing cells strongly reduced the toxic effects of α-synuclein with a significant marked increase in mean lifespan (11.19 ± 2.18 days *vs* 2.22 ± 0.31 days; 404% increase) (Figure 4A, Table 3). Although α-synuclein protein was still present 3 days after the treatment (Supplementary Figure 3), it was less localized to the plasma membrane, as shown by immunofluorescence analysis (Figure 4B, 4C) and cell fractionation (Figure 4D). These data suggest that a different localization of α-synuclein, rather than its protein clearance, could be responsible for the reduced toxicity in the presence of the bean extract.

**Table 3:** Mean and maximal lifespan of yeast cells bearing pYX242 empty vector or pYX242-SNCA plasmid grown in the absence or presence of 0.5% *V. unguiculata* extract.

**lifespan (days)**				
	**mean**		**maximal**	
**strain**	**cnt**	**0.5% E**	**cnt**	**0.5% E**
*[pYX242]*	9.96 ± 0.49	13.3 ± 1.13	>15	>15
*[pYX242-SNCA]*	2.22 ± 0.31	11.19 ± 2.18	14.21 ± 1.06	>15

The process of α-synuclein fibrillation was then investigated *in vitro* at two different concentrations of cowpea extract. The increase of ThT and ANS fluorescence emission intensity was used to quantify fibrils formation and conformational change of the protein with or without cowpea extract (Figure 4E, 4F). The presence of *V. unguiculata* extract led to a significant concentration-independent decrease of ThT fluorescence in the α-synuclein aggregation solution, with an increase of the lag time and a decrease of both β-sheet growth rate and final equilibrium levels (Figure 4E). In agreement with a nucleation-dependent polymerization model, α-synuclein exhibited a sigmoidal binding without cowpea extract (Figure 4E).

These evidences suggest that *V. unguiculata* extract significantly altered the amyloid aggregation pattern of α-synuclein. Moreover, the ANS binding fluorescence data indicate that the cowpea extract might increase the formation of α-synuclein species with minor solvent exposure of hydrophobic clusters, or it might decrease the binding of ANS to α-synuclein surfaces (Figure 4F). The morphology of α-synuclein aggregates was also studied by TEM analysis. After 24 h of aggregation, the protein, either alone or in the presence of cowpea extract, existed as globular micelle-like and prefibrillar assemblies (Figure 4G). After 72 h of aggregation in the absence of the extract, α-synuclein samples were mostly mature fibrils. Remarkably, the presence of cowpea extract enriched the samples with short fibrils covered by densely packed globular clusters (Figure 4G). Overall, these findings are consistent with an inhibitory effect of *V. unguiculata* extract on the formation of amyloid fibrils.

**Figure 5:**
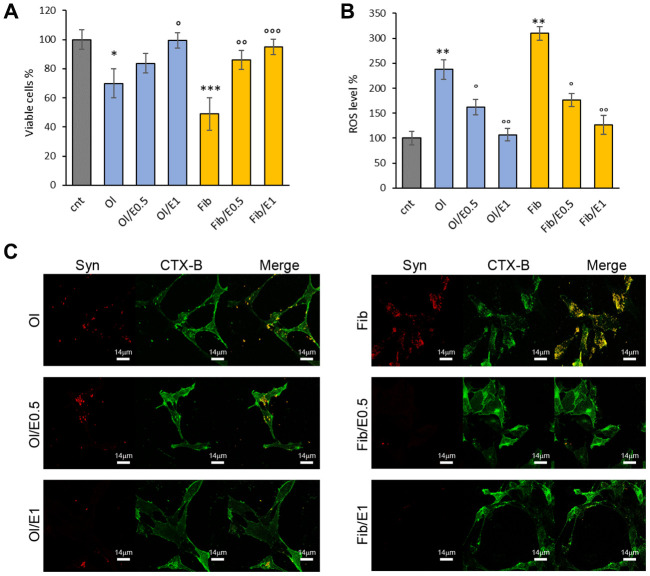
***V. unguiculata* extract reduces α-synuclein toxicity in neuroblastoma cells.** (**A**, **B**) SH-SY5Y cells were grown for 48 h in the absence (cnt) or presence of 5 μM α-synuclein solution obtained after 24 h (oligomers, Ol) and 72 h (fibrills, Fib) of aggregation, without or with extract at molar ratio protein:extract 1:0.5 (E0.5) and 1:1 (E1). (**A**) Cell viability assessed by MTT assay and (**B**) ROS level evaluated by DCFDA fluorescence intensity assays. *p<0.05; **p<0.01; ***p<0.001 *vs* untreated cells. °p<0.05; °°p<0.01 *vs* treated cells with α-synuclein aggregates oligomeric (Ol) and fibrillar (Fib) grown without extract. (**C**) Z-projection of SH-SY5Y cell images by α-synuclein immunostaining (red) and CTX-B plasma membrane staining (green). Scale bars are shown.

It has been reported that cytotoxicity of amyloidogenic species largely depends on their biophysical surface properties, which influences their reactivity with the cellular plasma membrane [42–44]. To assess α-synuclein toxicity, we performed MTT assays on the human neuroblastoma SH-SY5Y cell line exposed for 48 h to extracellular α-synuclein aggregates pre-formed *in vitro* in the absence or in the presence of cowpea extract. Coherently, α-synuclein obtained without extract supplementation exhibited the highest cytotoxicity: oligomers (Ol) and fibrils (Fib) showed about 70% and 50% viability, respectively (Figure 5A). In cells incubated with α-synuclein aggregates formed in the presence of the extract, toxicity was reduced and cell viability was about 83% with oligomers (Ol/E0.5) and 86% with fibrils (Fib/E0.5) increasing up to 100% and 95% at the highest concentration of the extract (Figure 5A). Accordingly, ROS level significantly decreased in cells exposed to oligomers and fibrils formed in the presence of cowpea extract (Figure 5B).

These data suggest that in the presence of *V. unguiculata* extract, α-synuclein aggregation is redirected into non-toxic aggregate species.

To further explore the potential mechanism for the protective effect of the cowpea extract, the interaction of α-synuclein aggregates with the plasma membrane of neuroblastoma cells was monitored by confocal microscopy. As previously reported [43, 44], a large number of α-synuclein oligomers or fibrils (stained in red) were bound to the cell membrane (stained in green) (Figure 5C). When cells were exposed to α-synuclein aggregates formed in the presence of cowpea extracts, the binding of both oligomers and fibrils to the cellular membranes was drastically reduced (Figure 5C).

In conclusion, these data show that the presence of cowpea extract during α-synuclein aggregation decreases the ability of the resulting aggregates to bind the plasma membrane and to raise ROS production and cytotoxicity.

### *Vigna unguiculata* extract reduces α-synuclein induced neurodegeneration in *Caenorhabditis elegans*

**Figure 6:**
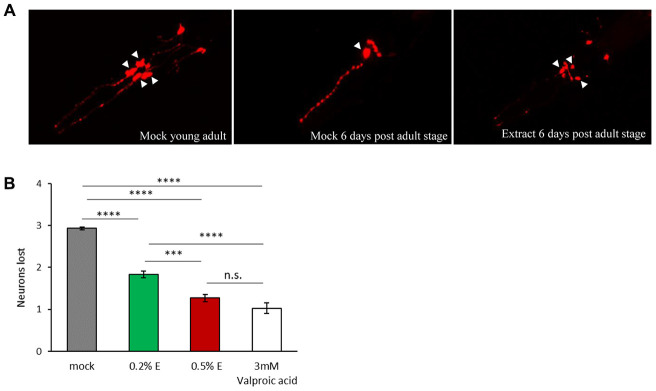
***V. unguiculata* extract is neuroprotective in a dose-dependent manner in a *C. elegans* model of α-synuclein toxicity.** (**A**) The four CEP neurons (indicated by arrowheads), expressing DsRed and human α-synuclein, are viable and with a wild type morphology in young adult animals cultivated in mock conditions (left panel); only one neuron is visible and viable after 6 days from adult stage cultivated in mock conditions (central panel); 0.5% *V. unguiculata* extract partially rescued the neurodegeneration after 6 days from adult stage (right panel). Anterior is to the left. (**B**) Quantification of dopaminergic neuron loss in human α-synuclein expressing animals grown with 0.2% and 0.5% of *V. unguiculata* extract. ***p<0.001, ****p<0.0001. The number of animals scored with mock is 95, with 0.2% extract is 103, with 0.5% extract is 94 and with 3 mM valproic acid 92.

In order to evaluate the neuroprotective effects of cowpea extract on a multicellular organism, we turned to the nematode *C. elegans.* The expression of human α-synuclein in *C. elegans* causes the age-dependent degeneration and death of the four cephalic dopaminergic neurons (CEP), a phenotype which can be easily scored using a red fluorescent marker expressed only in those neurons [45]. Consistent with previous reports, we observed an age-related decline in the number of fluorescent dopaminergic neurons expressing human α-synuclein (Supplementary Figure 4). Thus, we investigated the effects of *V. unguiculata* extract both at 0.2% and 0.5%, in 6-day adult animals (Figure 6A, 6B). While in mock treated animals a mean of 3 out of 4 CEP neurons died, in animals exposed to *V.* *unguiculata* extracts there was a partial rescue of neurodegeneration, with 2 neurons dying in 0.2% extract and only 1 in 0.5%. A similar effect was observed in animals treated with 3 mM valproic acid (positive control) [46].

Our results indicate that *V. unguiculata* extract protects CEP dopaminergic neurons from degeneration in a *C. elegans* model of α-synuclein toxicity, in a dose-dependent manner.

## DISCUSSION

Cowpea is considered as a source of health-promoting compounds, with a low fat and high protein content, as well as dietary fibers, phenolic compounds and minerals. Consumption of cowpea is associated with reduced risk of gastrointestinal disorders, cardiovascular diseases, hypercholesterolemia, obesity, diabetes and several types of cancer [47]. We now add new important health benefits of cowpea beans, *i.e*. their anti-aging and neuroprotective effects. Indeed, we show that *V. unguiculata* extract extends lifespan, in two different eukaryotic models, such as budding yeast and fruit flies (Figures 1, 3). The extension of longevity requires Snf1/AMPK pathway in yeast (Figure 2F) and induces the upregulation of two downstream proteins of the AMPK pathway, such as FOXO and SIRT1 in *Drosophila* (Figure 3) [48–50]. Strikingly, it has been reported that AMPK and SIRT1 are downregulated with aging and their pharmacological activation is necessary to increase longevity [5].

The anti-senescence properties of cowpea extract are strongly additive with caloric restriction (Supplementary Figure 1), the most effective non-genetic intervention delaying senescence [51], suggesting that *V. unguiculata* beans could display their best effect in terms of aging delay in a proper dietary regimen.

The strong neuroprotective features of cowpea extract are conserved in evolutionary distant eukaryotic systems. Indeed, *V. unguiculata* extract decreases α-synuclein toxicity in both yeast and neuroblastoma cells, as well as in a *C. elegans* PD model by partially rescuing the degeneration of cephalic dopaminergic neurons (Figure 6).

The anti-aggregation properties of cowpea on α-synuclein is clearly evident (Figure 4E–4G). Along similar lines, the extract decreases the localization of α-synuclein to cell membrane both in the yeast model, in which α-synuclein is intracellularly expressed (Figure 4A–4D), and in neuroblastoma cells, where α-synuclein is added to the medium (Figure 5), also in keeping with the minor solvent exposure of hydrophobic clusters detected by ANS on amyloid assemblies (Figure 4F). These results suggest that *V. unguiculata* extract decreases the neurotoxicity caused by the intracellular accumulation of α-synuclein aggregates and the cellular damage induced by oligomeric aggregates interacting with the cell membrane by displacing the toxic protein from the lipidic bilayer. Our data are in accordance with recent results showing that inhibition of α-synuclein binding to membranes reduces the toxicity of the protein both in worms and in mice [52, 53].

The aqueous extract of cowpea beans contains starch, amino acids, as well as several different proteins and peptides (Figures 1A–1C, 2A, 2B), while it is probably very poor in phenolic compounds. Although abundance of starch and proteins is generally considered negative from an aging point of view [10], the protection against senescence and neurodegeneration might be the result of a synergistic effect of different elements. Indeed, the nutrient combination of the extract rather than a single component might be responsible for the metabolic reprogramming, which leads to the longevity phenotype.

Although the identity of the active components in the extract remains to be investigated, the extraction process strongly mimics the way in which these beans are consumed. Therefore, the anti-aging and neuro-protecting compounds are likely to be conserved during the cooking process. Remarkably, it has been reported that cooking legumes in water increases the insoluble fiber content, protein quality and digestibility, although with a reduction of the content of vitamins and minerals [47]. Therefore, the use of cowpea beans should be encouraged and eventually the identification of the bioactive compounds could lead to the development of specific dietary supplements to support healthy aging and to delay neurodegeneration.

Any dietary intervention that has the potential of delaying the progression of age-related diseases could improve the quality of life of the aging population, inducing also an important impact on the economic implications of elderly on the society. Thus, *V. unguiculata* consumption in the global food chain is encouraging since our study suggests that cowpea beans supplementation can prevent age-related disorders.

Although data on bioactive compounds from cowpea are still poor, some reports indicate components like peptides may contribute to health benefits derived from cowpea [28]. Remarkably, several proteins identified by proteomic analysis are still uncharacterized (Figure 2A, 2B). We believe that additional work is necessary to discover the bioactive compounds in cowpea and their interactions to efficiently exploit them in foods, such as snacks and breakfast cereals by targeting benefits to immune function and health gut. Indeed, interesting data show that progression of PD has been frequently associated with dysbiosis of gut microbiota [54, 55].

In conclusion, considering the role of functional food in the management of age-related diseases, we strongly support the intake of *V. unguiculata* beans to reduce senescence, neuroinflammation and the extent of neurodegeneration.

## MATERIALS AND METHODS

### Extract preparation

*V. unguiculata*, *C. cajan* and *P. vulgaris* seeds were purchased from two markets (Kilombero and Arusha Central Market) in Arusha, Tanzania (3°22'0.01"S, 36°40'59.99"E). Seeds of each species were boiled for 1 h and left cool down for the subsequent hour. The treatment was performed to mimic the condition of consumption, as described in [56]. Then, seeds were incubated at 50°C overnight till dryness and grinded to obtain a fine powder. 2 g of seed dry powder were suspended in 50 ml of ultrapure MilliQ water. Then, pulses were extracted through a magnetic stirrer at 500 rpm for 5 minutes and centrifuged at 5000 g for 30 min. Supernatant was recovered and freeze-dried.

### Chemical and proteomic characterization of the extracts

### Starch content

Starch content was evaluated by the enzymatic assay Total Starch AOAC Method 996.1 1 and AACC Method 76.13 (Megazyme ®, Ireland). Briefly, 50 mg of extract were suspended in 200 μl of ethanol 80% v/v and 1 ml of 2 M KOH. Samples were magnetically stirred for 20 min at 4°C. Then, 4 ml of sodium acetate pH=3.8 were added, followed by the addition of 50 μl of α-amylase (8300 U/mL) and 50 μl of amyloglucosidase (AMG, 3300 U/ml). Samples were incubated for 30 min with intermittent mixing on a vortex mixer, then centrifuged for 10 min at 3000 rpm to recover the supernatant. In order to evaluate total starch content, a reaction mixture was prepared as follows in a quartz cell: 1 ml H_2_O, 25 μl of sample, 50 μl of a buffer solution pH = 7.6, 50 μl NADP+/ATP. The solution was incubated for 3 min at room temperature and then the absorbance was read at 340 nm against the blank. Then, 10 μl of a solution containing hexokinase (HK) and glucose-6-phosphate-dehydrogenase (G6PDH) was added. After an incubation of 5 min at room temperature, the absorbance was read against the blank again at 340 nm. Data are expressed as g of starch per 100 g of extract.

### Amino acids content

Amino acids were quantified through a HPLC-DAD method. A 1260 Infinity II LC System (Agilent, USA, 2018) was set up for the analysis. The calibration curve was made up using an amino acid mixed solution (Merck, Germany) in a concentration range between 0.078 mM and 1.25 mM. The column was an Agilent Poroshell HPH C18 (100 x 4.6 mm, 2.7 μm) coupled with a guard column (AdvanceBio Oligo 4.6 x 5 mm, 2.7 μm) and it was kept at 40°C. Mobile phases were: A - 10 mM Na_2_HPO_4_ pH=8.2 and B - Acetonitrile:Methanol:Water 45:45:10. The elution program was the following (%B): 0-0.35 min 2%, 13.4 min 57%, 13.5 min 100%, 15.7 min 100%, 15.8 min 2%, 18 min end. Flow rate was constant at 1.5 ml/min. All solvents were HPLC grade, whereas the buffer, solutions and samples were pre-filtered with a 0.22 μm filter. OPA (o-Phthaldialdehyde reagent, Merck, Germany) was chosen as derivatizing agent acting as a fluorophore. Injection volume was 10 μl. The signal used to visualize the fluorescence was set at 338 nm bandwidth 10 nm with a reference wavelength of 390 nm bandwidth 20 nm. All data were displayed and analyzed on Agilent ChemStation software. Data are expressed as g of amino acids per 100 g of extract.

### Proteins content

Total protein content was evaluated by using the Bradford assay as follows: 1 ml of 50% Coomassie-Brilliant Blue Bradford reagent (ThermoFisher, USA) was incubated at room temperature with 2 μl of extract of known concentration for a minute. Absorbance was read against blank at 595 nm and fitted on a calibration curve made up with BSA (Bovine Serum Albumin) in a range between 0 and 6 mg/ml. Data are expressed as g of protein per 100 g of extract.

### GC/MS analysis

Before the GC/MS analyses all samples were subjected to a derivatization process, as described below. About 5 mg of each sample were accurately weighed, suspended in 50 μl of 2wt% methoxylamine hydrochloride in pyridine and incubated for 90 min at 37°C. Then, 80 μl of MBDSTFA (N-methyl-N-ter-butyldimethylsilyl-trifluoroacetamide)+1% TBDMCS (tert-butyldimethylchlorosilane) were added and the samples were incubated at 60° C for 30 min. After incubation at room temperature overnight, the samples were analyzed by using a ISQ™ QD Single Quadrupole GC-MS (Thermo Fisher) equipped with a VF-5ms (30 m x 0.25 mm i.d. x 0.25 μm; Agilent Technology). Injection volume: 1 μl. Oven program: 100° C for 2 min; then 6° C/min to 280° C for 15 min; Run Time 42 min. Helium was used as the gas carrier. SS Inlet: Mode Splitless. Inlet temperature: 280° C. Flow 1.0 ml/min. MS transfer line: 270° C. Ion source: 250° C. Ionization mode: electron impact: 70 eV. Acquisition mode: full scan. In order to compare the composition of the extracts, for each analyte identified by GC/MS a target ion (m/z) was extracted by the TIC and the corresponding area was calculated. Supplementary Table 2 reports for each analyte the corresponding target ion used.

### Proteomic analysis

The extract was reduced, derivatized and digested with trypsin (protein: protease ratio 20:1) as described in [57] before MS/MS analysis.

Peptides separation was achieved on a Thermo Easy-nLC 1000, with a linear gradient from 95% solvent A (2 % ACN, 0.1% formic acid) to 30% solvent B (80% acetonitrile, 0.1% formic acid) over 60 min, from 30 to 60% solvent B in 5 min and from 60 to 100% solvent B in 2 min at a constant flow rate of 0.25 μl/min, with a single run time of 75 min. MS data were acquired on a Thermo Q-Exactive-HF, with a data-dependent top 15 method, the survey full scan MS spectra (300-1650 m/z) were acquired in the Orbitrap with 60000 resolution, AGC target 3e6, IT 20 ms. For HCD spectra resolution was set to 15000, AGC target 1e5, IT 80 ms; normalized collision energy 28 and isolation width of 1.2 m/z.

Raw label-free MS/MS files from Thermo Xcalibur software (version 4.1) [57] were analyzed using Proteome Discoverer software (version 2.2, Thermo Fisher Scientific) and searched with Sequest algorithm against the proteome of NCBI Phaseoleae (release 05/08/2019). The minimum required peptide length was set to 6 amino acids with carbamidomethylation as fixed modification, Met oxidation and Arg/Gln deamidation as variable modifications.

The mass spectrometry proteomic data have been deposited to the ProteomeXchange Consortium via the PRIDE [58] partner repository with the dataset identifier PXD017716.

### Yeast methods

### Yeast strains and media

The yeast strains used in this paper are listed in Supplementary Table 3. Cells were grown at 30°C in minimal medium containing 2% glucose as a carbon source and 0.67% yeast nitrogen base without amino acids, supplemented with 50 mg/l of required amino acids and bases for which the strains were auxotrophic. The natural extracts were dissolved in the medium at a concentration of 0.2% or 0.5% and filtered through 0.22 μm filters.

### Chronological lifespan experiments (CLS)

Cell cultures were grown in liquid medium until mid-late exponential phase and then inoculated into flasks containing medium in the presence or absence of the natural extracts (0.2% or 0.5% as indicated in each experiment). Survival was assessed by propidium iodide staining (PI) at the indicated time points with the Cytoflex cytofluorimeter (Beckman Coulter) and analyzed with the Cytoflex software. For some experiments, survival was also confirmed by colony-forming units (CFUs) after 2 days of incubation at 30°C on YEPDA agar plates.

### Protein extraction, cell fractionation and immunoblotting

Equal amounts of cells were collected and quenched using TCA 6% and lysed in lysis buffer (6M UREA, 1% SDS, 50 mM Tris-HCl pH7.5, 5 mM EDTA). The cytoplasmic-membrane fractionation experiment was conducted using the MEM-PER kit (Thermo), following the manufacturer’s instructions on yeast spheroplasts. Western blot analysis was performed using anti-Synuclein antibody (1:1000, Sigma Aldrich), anti-Pgk1 antibody (1:1000, Molecular Probes, used as loading control and cytoplasmic marker) and anti-Pma1 antibody (1:1500, Abcam, used as membrane marker).

### Glucose consumption assay

Extracellular levels of glucose were evaluated on growth media of wt cells exponentially growing in the absence or presence of 0.5% cowpea extract, using the Megazyme D-glucose-HK assay kit, following the manufacturer's instructions, using an EnSight Plate Reader (Perkin Elmer).

### Fluorescence microscopy on yeast

*In situ* immunofluorescence was performed on formaldehyde-fixed cells and carried using α-synuclein immunostaining (1:2000, Sigma Aldrich) followed by indirect immunofluorescence using rhodamine-conjugated anti-rabbit antibody (1:1000, Pierce Chemical Co). Digital images were taken with a Nikon DS-Qi MC camera mounted on a Nikon Eclipse 600 and controlled by the NIS elements imaging software (Nikon) with an oil 100X 0.5-1.3 PlanFluor oil objective (Nikon).

### In vitro aggregation of α-synuclein

α-synuclein was expressed in *Escherichia coli* BL21(DE3) cells transformed with the pET28b/α-synuclein plasmid. The recombinant protein was expressed and purified according to a previously described procedure [43] and further purified by RP-HPLC. The identity and purity of the eluted material were assessed by mass spectrometry**.** Protein samples (250 μM), filtered through a 0.22 μm pore-size filter (Millipore, Bedford, MA, USA) were incubated at 37°C in 20 mM sodium phosphate buffer, pH 7.4 up to 3 days under shaking at 900 rpm with a thermo-mixer in the absence or in the presence of extract by using molar protein/substance ratios of 1:0.5 (E0.5) and 1:1 (E1). Oligomer-enriched or fibril-enriched sample were prepared by incubating a-synuclein for 24 h or 72 h, respectively.

### ThT assay

The ThT binding assay was performed according to LeVine [59], using a 25 μM ThT solution in 20 mM sodium phosphate buffer, pH 7.0. Each sample, diluted at a final concentration of 6.25 μM, was transferred into a 96-well half-area, low-binding, clear bottom (200 μl/well) and ThT fluorescence was read at the maximum intensity of fluorescence of 485 nm using a Biotek Synergy 1H plate reader; buffer fluorescence was subtracted from the fluorescence values of all samples. In controls experiments, a significant interference of the highest concentrations of cowpea extract on ThT fluorescence was observed, so the two molar ratio protein:extract with lowest fluorescence interference were selected (Supplementary Figure 5).

### ANS assay

Samples containing aggregating a-synuclein with and without cowpea extract at 250 μM were investigated for their ability to bind 8-anilinonaphthalene-1-sulfonic acid (ANS; Sigma Aldrich, Saint Louis, MO, US). 5 μl of each samples at different times of aggregation was transferred into a 96-well half-area, low-binding, clear bottom (200 μl/well), and ANS (50 μM) fluorescence intensity was read at the binding intensity of fluorescence of 480 nm in a Biotek Synergy 1H plate reader; buffer fluorescence was subtracted from the fluorescence values of all samples. In control experiments, a significant interference of the highest concentrations of the extract on ANS binding fluorescence was observed, so we selected the two molar ratios protein:extract with the lowest fluorescence interference (Supplementary Figure 5).

### Transmission electron microscopy (TEM) imaging

5 μl aliquots of α-synuclein aggregated in the presence or in the absence of cowpea extract were withdrawn at different aggregation times, loaded onto a formvar/carbon-coated 400 mesh nickel grids (Agar Scientific, Stansted, UK) and negatively stained with 2.0% (w/v) uranyl acetate (Sigma-Aldrich). The grid was air-dried and examined using a JEM 1010 transmission electron microscope at 80 kV excitation voltage.

### Cell culture methods

### Cell culture

SH-SY5Y human neuroblastoma cells were cultured at 37 °C in complete medium (50% HAM, 50% DMEM, 10% fetal bovine serum, 3 mM glutamine, 100 units/ml penicillin and 100 μg/ml streptomycin), in a humidified, 5% CO_2_ incubator.

### MTT assay

Cell viability was assessed by the MTT assay optimized for the SH-SY5Y cell line. Briefly, SH-SY5Y cells were seeded into 96-well plates at a density of 10000 cells/well in fresh complete medium and grown for 24 h. Then, cells were exposed for 48 h to 5 μM α-synuclein obtained at different times of aggregation in the presence or in the absence of the *Vigna unguiculata* extract. Cells were also treated with the corresponding concentrations of extract used in the aggregation of α-synuclein and the viability resulted similar to that of untreated control cells. After 48 h of incubation, the culture medium was removed and cells were incubated for 1 h at 37°C in 100 μl serum-free DMEM without phenol red, containing 0.5 mg/ml MTT. Then, 100 μl of cell lysis solution (20% SDS, 50% N,N-dimethylformamide) was added to each well and samples were incubated at 37°C for 2 h to allow complete cell lysis. Absorbance values were measured using iMARK microplate reader (Bio-Rad) at 595 nm. Final absorption values were calculated by averaging each sample in triplicate after blank subtraction. Statistical analysis of the data was performed by using one-way analysis of variance (ANOVA).

### ROS determination

Intracellular reactive oxygen species (ROS) were determined using the fluorescent probe 2’,7’–dichlorofluorescein diacetate, acetyl ester (CM-H2 DCFDA; Molecular Probes), a cell-permeant indicator for ROS that becomes fluorescent upon removal of the acetate groups by cellular esterases and oxidation. SH-SY5Y cells were plated on 96-well plates at a density of 10000 cells/well and exposed for 48 h to the α-synuclein samples. Then, 10 μM DCFDA in DMEM without phenol red was added to each well. The fluorescence values at 538 nm were detected after 30 min by Fluoroscan Ascent FL (Thermo-Fisher). Cells were also treated with the corresponding concentrations of extract used in the aggregation of α-synuclein and ROS levels resulted similar to that of untreated control cells. Statistical analysis of the data was performed by using one-way analysis of variance (ANOVA).

### Confocal imaging

Subconfluent SH-SY5Y cells grown on glass coverslips were exposed for 48 h to 5 μM (monomer concentration) α-synuclein aggregates grown in the presence or in the absence of cowpea extract at different molar ratios (1:0.5, E0.5; 1:1, E1). Cell membrane labelling was performed by incubating the cells with 10 ng/ml Alexa Fluor 488-conjugated CTX-B (Cholera toxin B-subunit) in cold complete medium for 30 min at room temperature. Then, cells were fixed in 2.0% buffered paraformaldehyde for 6 min and permeabilized by treatment with a 1:1 acetone/ethanol solution for 4 min at room temperature, washed with PBS and blocked with PBS containing 0.5% BSA and 0.2% gelatin. After incubation for 1 h at room temperature with rabbit anti-synuclein polyclonal antibody (1:600 in blocking solution), the cells were washed with PBS for 30 min under stirring and then incubated with Alexa Fluor 568-conjugated anti-rabbit secondary antibody (Molecular Probes) diluted 1:100 in PBS. Finally, cells were washed twice in PBS and once in distilled water to remove non-specifically bound antibodies. Digital images were taken with a confocal Leica TCS SP8 scanning microscope (Leica, Mannheim, Ge) equipped with a HeNe/Ar laser source for fluorescence measurements. The observations were performed using a Leica HC PL Apo CS2 X63 oil immersion objective.

### *Drosophila melanogaster* methods

### Fly husbandry and supplementation and longevity assay

Wild type *Drosophila melanogaster* (Canton S) was kindly provided by Dr Daniela Grifoni (University of Bologna, Italy). Flies were maintained at constant temperature (25°C) and humidity (60%) with a 12/12 h light–dark cycle. Flies were reared on Formula 4-24 ® media (Carolina Biological, Burlington, NC, USA). The composition of this diet, as indicated by the manufacturer, is as follows: oat flour, soy flour, wheat flour, other starches, dibasic calcium phosphate, calcium carbonate, citric acid, niocinamide, riboflavin, sodium chloride, sodium iron pyrophosphate, sucrose, thiamine, mononitrate, brewer's yeast, emulsifier preservatives, mold inhibitor, food coloring. The Formula 4-24 diet requires separate application of yeast pellets (*Saccharomyces cerevisiae*) and saturation of this dry media mixture with water. After eclosion, males and females emerged within 1-2 day were allowed to mate freely for two days before female separation into vials containing 1 g Formula 4-24 Instant Drosophila Medium (Carolina) soaked with 4 ml water containing 0.5% or 0.2% bean extract. A total of 20 flies were placed in each vial.

Female flies emerging within a 2-day period were collected under FlyNap (Carolina) anaesthesia. A total of 600 fruit flies were divided into 3 groups: control group, flies supplemented with 0.2% bean extract and flies supplemented with 0.5% bean extract. Flies were transferred into vials containing fresh food every 2-3 days and the number of living flies was counted. This was repeated until all flies had died. Kaplan-Meier survival curves were generated for lifespan assessment.

### Measurement of Drosophila body weights

Changes in body weights were used as an indicator of the food intake. Flies were fed on standard diet with and without bean extract for 30 or 45 days. For each condition (0.2% bean extract supplementation and control), five vials containing 20 flies/vial were counted.

Flies in each group were anesthetized by FlyNap (Carolina) and then weighed on a balance. The mean body weights of the flies in each group were calculated.

### Gene expression analysis

Total RNA was extracted from the whole bodies of either 30 days or 45 days old flies belonging to the 0.2% group by using RNeasy Mini Kit (QIAGEN GmbH, Hilden, Germany). The 0.2% supplementation has been chosen because it was the one able to significantly increase lifespan in *Drosophila*. All the experiments were performed in triplicate. The yield and purity of the RNA were measured using NanoVue Spectrophotometer (GE Healthcare, Milano, Italy). Only samples with density ratios A260/A280>1.8 were used. cDNA was obtained by reverse transcribing mRNA starting from 1 μg of total RNA using iScript cDNA Synthesis Kit (BIO-RAD, Hercules, CA, USA), following the manufacturer's protocol. The subsequent polymerase chain reaction (PCR) was performed in a total volume of 10 μl containing 2.5 μl (12.5 ng) of cDNA, 5 μl SsoAdvanced Universal SYBR Green Supermix (BIO-RAD), 2 μl of dH20 RNA free and 0.5 μl (500 nM) of each primer. The primers used are reported in Supplementary Table 4 and RPL32 was used as reference gene.

### Protein extraction and immunoblotting

Proteins were isolated from the whole bodies of either 30- or 45-day old flies. Proteins were homogenized using lysis buffer (7 M urea, 2 M thiourea, 4% CHAPS, 60 mM dithiothreitol (DTT), 0.002% bromophenol blue) and centrifuged at 12000 g at 4 °C for 5 min. The supernatant was collected and mixed with Sample Buffer, Laemmli 2× Concentrate (Sigma Aldrich). Proteins were then loaded onto 4-20% SDS-PAGE gels followed by transfer onto nitrocellulose membranes and immunoblotted with appropriate antibodies. Anti-Sirt1 (1:1000; Cell Signaling Technology, Beverly, MA), anti-dFoxO (1:1000, Covalab, Villeurbanne, France) and anti-β-actin (1:2000, Invitrogen Carlsbad, CA, USA) antibodies were used as primary antibodies. The HRP-conjugated anti-mouse IgG and anti-rabbit IgG antibodies were employed as the secondary antibodies (1:10000; Cell Signaling Technology). Targeted proteins were visualized using Clarity^TM^ Western ECL Substrate (BIO-RAD). Densitometric analysis of specific immunolabeled bands was performed using ImageJ software.

### Statistical analysis

Each experiment was performed at least three times, and all values are represented as means ± SD. One-way ANOVA was used to compare differences among groups followed by Dunnett’s (Prism 5; GraphPad Software, San Diego, CA). Values of p<0.05 were considered statistically significant. Survival curves were prepared by Kaplan-Meier survival analysis and analyzed using the OASIS2 software [60].

### *Caenorhabditis elegans* methods

### C. elegans strains and treatment with V. unguiculata extract

Standard procedures for *C. elegans* strain maintenance were followed [61]. The strain used in this study, JZF142 [*pdat-1::hαSyn; pdat-1::DsRed*], was kindly provided by Prof. J. Feng (Case Western Reserve University, US) [45]. The *C. elegans* strain was grown on Nematode Growth Medium (NGM) containing agar, seeded with *E.coli* OP50 at 20°C. Lyophilized extract from *V. unguiculata* was solubilized in sterile distilled water at two dilutions, 2% or 5% w/V, and sterilized with 0.22μm filter. α-synuclein expressing animals were exposed to the following treatments: 0.2% or 0.5% of *V. unguiculata* extract, water as negative control (mock) and 3 mM valproic acid (VA) as positive control [46]. *C. elegans* animals at L4 developmental stage were transferred into 12-well plates with NGM agar containing the different conditions as quadruplicates and allowed to become adults and lay eggs. After 14 hours the adults were discarded and the synchronized F1 progeny was allowed to grow in the presence of chronic treatments. F1 animals have been transferred every 3 days on new plates with treatment, until the day of analysis, to maintain them well fed and separated from the next generation.

The morphology of the four cephalic dopaminergic neurons (CEP neurons) in the F1 treated animals was scored at 6 days post adult stage. The neurodegeneration analysis was performed also on untreated animals at young adult and at 6 days post-adult stage. Animals were mounted and anesthetized with 0.01% tetramisole hydrochloride on 4% agar pads. The neurodegeneration analysis was performed using Zeiss Axioskop microscope (Carl Zeiss). All images were obtained using a Leica SP2 confocal laser scanning microscope (Leica). The spectra used for imaging DsRed were: λ excitation=543 nm and λ emission=580-630 nm. GraphPad Prism software was used for statistical analysis. The statistical significance was determined using Mann Whitney test or One-way ANOVA with Kruskal–Wallis post-test. Data are reported as averages of multiple observations ± SEM.

## Supplementary Material

Supplementary Figures

Supplementary Tables
