# Supplementary material for: Protective effect of Vigna unguiculata extract against aging and neurodegeneration
Source: Aging (Albany NY). 2020 Oct 5;12(19):19785–808. doi: 10.18632/aging.104069 (PMC7732273; doi:10.18632/aging.104069)
Supplement: Supplementary Figures [file aging-12-104069-s001..pdf]

SUPPLEMENTARY FIGURES

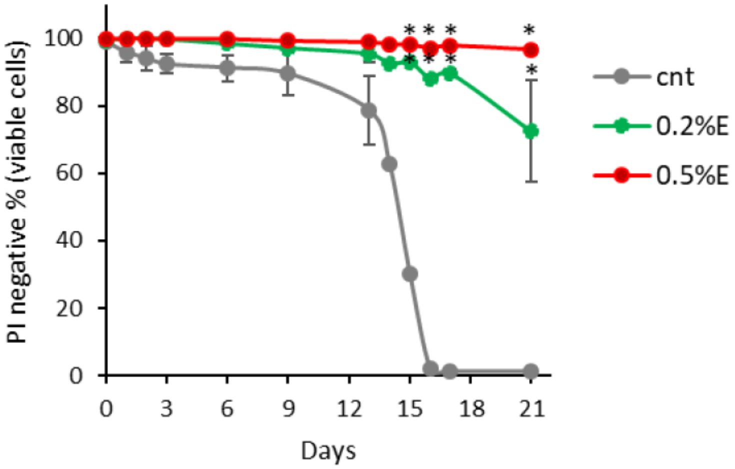

Supplementary Figure 1. The effect of cowpea extract is synergistic with caloric restriction. CLS of yeast wt cells grown in SD medium containing 0.5% glucose in the absence or presence of 0.2% or 0.5% extract of *Vigna unguiculata*. *p<0.05 relative to control cells.

A

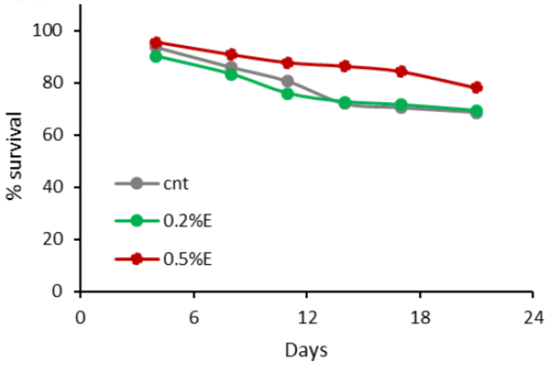

B

Condition	P-value
CTRL_F v.s. 0.5% bean_F	0.0032
CTRL_F v.s. 0.2% bean_F	0.9985
0.5% bean_F v.s. 0.2% bean_F	0.0032

Supplementary Figure 2. Survivorship of adult female *D. melanogaster*. (**A**) Flies have been supplemented with 0.2 and 0.5% cowpea extract lifelong. Data are presented as percentage survival of flies as function of time (in days). (**B**) Analysis of the survivorship data with log-rank test using the online application for the survival analysis of life-span assays OASIS.

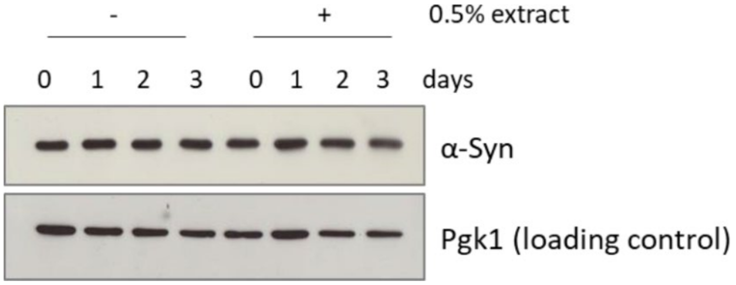

Supplementary Figure 3. α-synuclein level is unchanged but it is differently localized in the presence of cowpea extract in yeast cells. Western blot analysis is using anti-synuclein antibody in wt [pYX242-SNCA] cells after 5h, 1 day, 2 days, 3 days treatment with 0.5%*V. unguiculata* extract. Pgk1 was used as loading control.

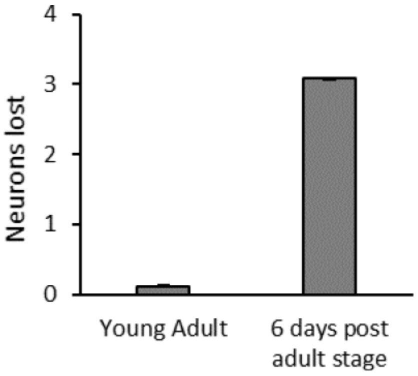

Supplementary Figure 4. α-synuclein causes age-related dopaminergic neurons lost in *C.elegans*. Number of nonvisible CEP dopaminergic neurons in human α-synuclein expressing strain. In a wild type strain four CEP neurons expressing DsRed are always visible (not shown). In young adults almost no neurodegeneration is visible, while 6 days after adult stage a mean of 3 neurons is lost (p<0.0001 Mann-Whitney non parametric test). Error bars represent the SEM for two independent experiments. The number of young adult animals scored is 100, while after 6 days post adult stage is 91.

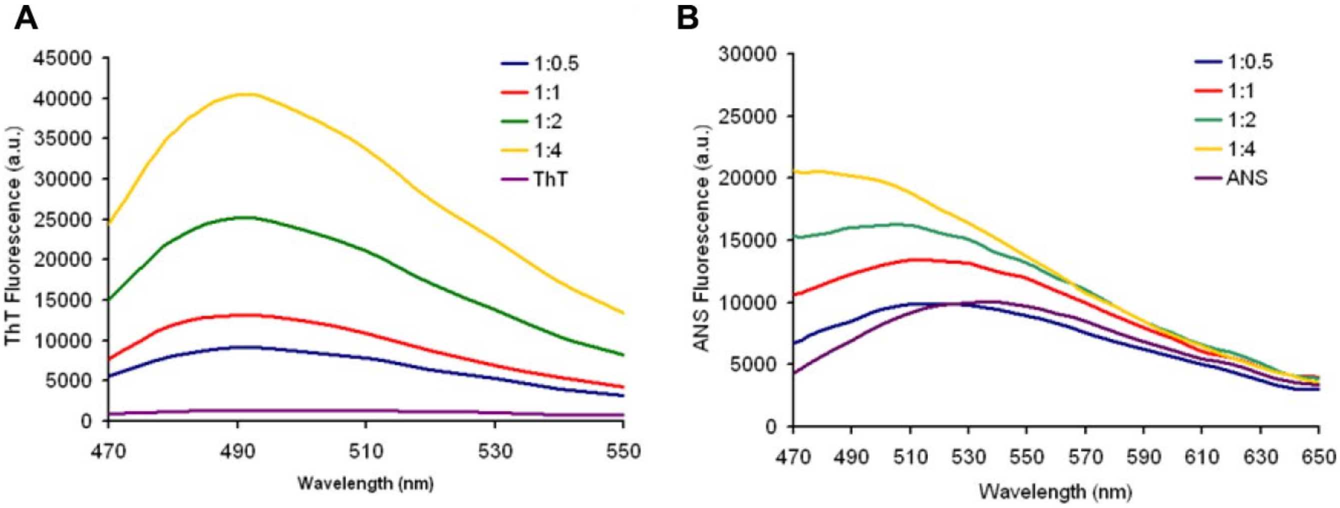

Supplementary Figure 5. Extract interferences on ThT and ANS fluorescence.
