# Supplementary material for: Protective effect of Vigna unguiculata extract against aging and neurodegeneration
Source: Aging (Albany NY). 2020 Oct 5;12(19):19785–808. doi: 10.18632/aging.104069 (PMC7732273; doi:10.18632/aging.104069)
Supplement: Supplementary Tables [file aging-12-104069-s002..pdf]

SUPPLEMENTARY TABLES

Supplementary Table 1. Metabolite content in extracts of *P. vulgaris*, *C. cajan* and *V. unguiculata*, analysed by GC/MS. Relative amounts of each metabolite are referred to *Phaseolus vulgaris* content, which was set to 1.

	Phaseolus vulgaris	*Cajanus cajan*	*Vigna unguiculata*
**aa and bases**			
glycine	1.00	1.07	2.66
L-leucine	1.00	0.49	2.90
L-threonine	1.00	0.00	10.74
L-alanine	1.00	0.80	2.72
L-proline	1.00	34.35	3.94
L-asparagine	1.00	0.63	52.44
L-aspartic acid	1.00	1.55	1.05
hypoxanthine	absent	absent	present
D-pyroglutamic acid	1.00	3.93	3.17
methylthiouracil	1.00	1.11	2.40
**glycolysis/fermentation and TCA**			
citric acid	1.00	1.90	2.25
malic acid	1.00	0.11	0.17
lactic acid	1.00	0.98	4.60
**fatty acids**			
glycerol	1.00	0.71	3.28
linolealidic acid	1.00	5.33	8.41
linolenic acid	1.000	0.162	2.663
stearic acid	1.000	0.968	1.342
palmitic acid	1.000	0.779	1.209
**other organic acids**			
D-pipecolic acid	1.00	0.78	absent
2-pentenedioic acid	1.00	1.34	1.14
2-thiobarbituric acid	1.00	0.66	0.29
L-dihydroorotic acid	1.00	0.62	0.84
glycolic acid	1.00	1.13	1.01
D-pipecoli acid	1.00	0.69	0.00
hydracrylic acid	1.00	6.98	1.05
butenoic acid	1.00	2.46	9.71
2-pyrrolidinone-5-carboxylic acid	1.00	1.18	0.43
**others**			
methyl-amino acetate	1.000	0.013	0.218
acetamide	1.000	0.577	18.533
triazol-3-amine	1.000	0.910	2.026
maltol	1.000	1.291	1.125
2-mercaptophenol	1.000	1.108	1.276
urea	1.000	very low	3.444

Supplementary Table 2. Identified analytes by GC/MS and target ion used to measure the peak area.

Analytes	Target ion (m/z)
glycine	246
L-leucine	200
L-threonine	404
L-alanine	232
L-proline	184
L-asparagine	417
L-aspartic acid	418
hypoxanthine	307
D-pyroglutamic acid	300
methylthiouracil	313
Citric acid	459
Malic acid	419
DL-glyceraldehyde	115
Lactic acid	261
glycerol	377
Linolealidic acid	337
Linolenic acid	335
Stearic acid	341
Palmitic acid	313
D-pipecolic acid	186
2-pentendioic acid	315
2-thiobarbituric acid	429
L-dihydroorotic acid	443
Glycoli acid	247
2-pipecoli acid	186
Hydroacrylic acid	261
Butenoic acid	289
2-butenoic acid	273
2-pyrrolidinone-5-carboxylic acid	186
Methyl-amino-acetate	146
acetamide	116
Triazol-3-amine	213
maltol	183
2-mercaptophenol	297
urea	231

Supplementary Table 3. Yeast strains used in this study.

Strain	Genotype	Source
wt	*BY4742 MATα his3Δ1 leu2Δ0 lys2Δ0 ura3Δ0*	Euroscarf
snf1Δ	*BY4742 MATα his3Δ1 leu2Δ0 lys2Δ0 ura3Δ01 snf1::HPH*	This study
atg1Δ	*BY4742 MATα his3Δ1 leu2Δ0 lys2Δ0 ura3Δ01 atg1::KanMX*	This study
ras2Δ	*BY4742 MATα his3Δ1 leu2Δ0 lys2Δ0 ura3Δ01 ras2::KanMX*	This study
tor2Δ	*BY4742 MATα his3Δ1 leu2Δ0 lys2Δ0 ura3Δ01 tor2::KanMX*	This study
wt [empty]	*BY4742 MATα his3Δ1 leu2Δ0 lys2Δ0 ura3Δ0 [pYX242]*	This study
wt [αSyn]	*BY4742 MATα his3Δ1 leu2Δ0 lys2Δ0 ura3Δ0 [pYX242-SNCA]*	This study

Supplementary Table 4. List of primers for real-time PCR.

Gene	5'-Forward-3'	5'-Reverse-3'
Sirt1	CATTATGCCGCATTTCGCCA	GAAGGTGTTCACTGAGGCCA
Foxo	AGGCTGACCCACACAGATAAC	GGCTCCACAAAGTTTTCGGG
Notch	CGCTTCCTGCACAAGTGTC	GCGCAGTAGGTTTTGCCATT
HO	ATGTCAGCGAGCGAAGAAACA	TGGCTTTACGCAACTCCTTTG
Trxr	TGGATCTGCGCGACAAGAAAG	GAAGGTCTGGGCGGTGATTG
RPL32	GCCCACCGGATTCAAGAAGT	CTTGCGCTTCTTGGAGGAGA
